# Factors associated with postoperative quality of life in patients with spinal metastases from lung cancer

**DOI:** 10.1097/MD.0000000000045359

**Published:** 2025-11-07

**Authors:** Yan Li, Fangzhi Liu, Zhongjun Liu, Xiaoguang Liu, Hua Zhou, Xiao Liu, Yanchao Tang, Panpan Hu, Feng Wei

**Affiliations:** a Department of Orthopedics, Peking University Third Hospital, Beijing, China; b Beijing Key Laboratory of Spinal Disease Research, Beijing, China.

**Keywords:** lung cancer, quality of life, spinal cord compression, spinal metastases

## Abstract

The management of spinal metastases presents a significant challenge for spine surgeons, especially in cases of lung cancer, which is associated with the poorest prognosis among primary cancer types. This study aimed to evaluate the postoperative quality of life of patients with symptomatic spinal metastases from lung cancer and identify clinical factors associated with improved outcomes regarding quality of life. This was a retrospective review of a prospectively maintained database from November 2009 to November 2020, including 128 patients who underwent surgery for symptomatic spinal metastases from lung cancer. The primary outcome was the change in Karnofsky Performance Status (KPS) at 1 week and 6 months post-operation. Patients were dichotomized into an “improvement” group (KPS increased) and a “non-improvement” group (KPS stable or decreased). Binary logistic regression was used to identify independent preoperative factors associated with KPS improvement. A total of 72 males and 56 females, with a mean age of 60 ± 10 years, were enrolled in the current study. Male sex was associated with improved quality of life in the short-term post-operation (odds ratio [OR] = 0.42, 95% confidence interval [CI] [0.716–0.962]). Conversely, the number of total bone metastatic sites was negatively associated with short-term improvements in quality of life (OR = 3.66, 95% CI [1.55–8.67]). Additionally, a higher number of total bone metastatic sites was linked to reduced long-term improvements in quality of life, with an OR of 1.94 and a 95% CI of [1.05–3.59]. The number of bone metastasis sites is closely associated with postoperative quality of life in patients with spinal metastases from lung cancer. Careful patient selection is crucial and has the potential to significantly enhance the quality of life for these vulnerable individuals with limited life expectancy.

## 1. Introduction

The spine is the third most common site of metastases, and the treatment of spinal metastases is mostly palliative, with the goals of providing pain relief, maintenance or recovery of neurologic function, local durable tumor control, spinal stability, and improved quality of life. It is estimated that metastatic spinal cord compression occurs in 5% to 10% of cancer patients (most commonly from breast, prostate, and lung cancers) and in up to 40% of patients who have preexisting non-spinal bone metastases.^[1,2]^ Approximately 50% to 70% of patients with solid cancers will develop spinal metastases during their illness.^[3]^ Due to advancements in cancer treatment that have extended patient survival, The management of spinal metastases has become an increasing concern for spine surgeons.

**Figure 1.**
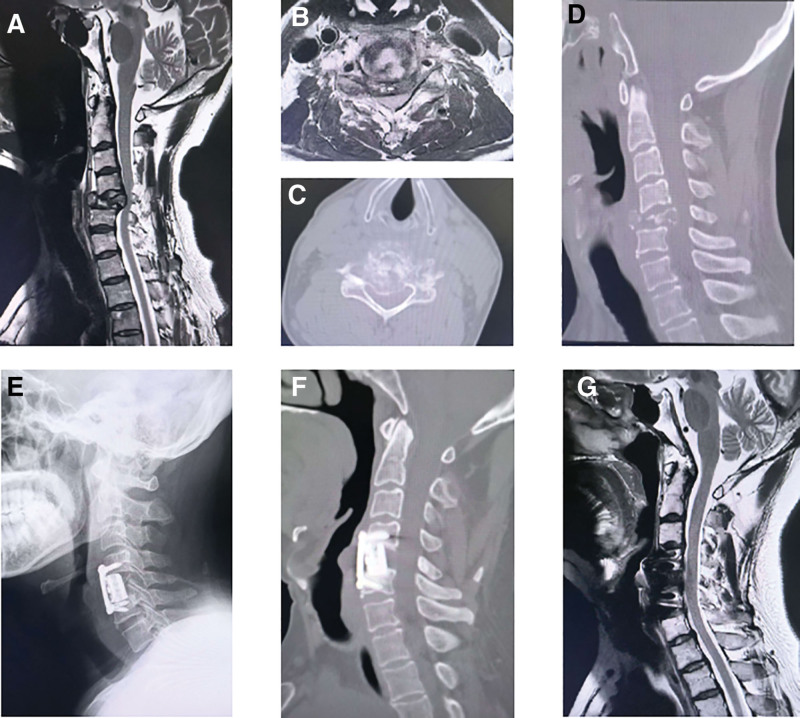
Illustrative case presentation. A 51-year-old male patient with cervical metastases from lung cancer presented with severe neck pain, numbness in both hands, and unsteady gait lasting for 2 weeks. MRI (A and B) and CT (C and D) imaging revealed an osteolytic metastatic lesion at C5 with a pathological fracture and spinal cord compression. The patient underwent anterior cervical corpectomy, decompression, and fusion (ACCF) combined with internal fixation to restore cervical alignment and decompress the spinal cord (E). Within 1 month, stereotactic body radiation therapy (SBRT) was administered, followed by chemotherapy and immunotherapy. At the 7-month follow-up, the patient experienced significant relief from neck pain and reported an improved quality of life. Imaging studies showed effective control of the cervical spine tumor (F and G). CT = computed tomography, MRI = magnetic resonance imaging.

Given the significant variability in clinical and radiological presentations, treatment strategies for spinal metastases should ideally be determined through a multidisciplinary approach. This ensures that each patient receives the most appropriate care to preserve or improve their quality of life.^[4]^ Among the available treatment options, surgery has proven effective for pain relief, spinal cord decompression, and stabilization of affected vertebrae.^[5]^ This concern is especially relevant for patients with limited life expectancy, where the risks of surgery may outweigh its potential benefits.^[6]^ As a result, accurately assessing a patient’s life expectancy is critical when considering surgical options for spinal metastases. The Tokuhashi score is widely used to evaluate these patients’ prognoses.^[7]^ Other scoring systems also support the notion that the prognosis for spinal metastases originating from lung cancer is unfavorable.^[8]^ While established scoring systems like the Tokuhashi score are widely used to predict survival, they do not specifically address the likelihood of functional improvement. This represents a critical knowledge gap, as the primary goal of palliative surgery in this end-stage population is often to improve or maintain quality of life, not just extend it. Simply surviving a surgery is not a successful outcome if the patient’s functional status is not improved. Therefore, the specific purpose of this study was to identify preoperative clinical and radiological factors that are associated with an improved postoperative quality of life, defined by functional gain, in patients undergoing surgery for spinal metastases from lung cancer. An illustrative case is presented in Figure 1.

## 2. Materials and methods

### 2.1. Participants

This study was a retrospective review of a prospectively maintained institutional database. From November 2009 to November 2020, a total of 146 patients with symptomatic spinal metastases secondary to lung cancer were recruited for the study after undergoing palliative surgery at the Department of Orthopedics, Peking University Third Hospital, a tertiary referral spinal unit.

The inclusion criteria for this study were as follows: patients with a histopathologically confirmed diagnosis of lung cancer; radiologically confirmed symptomatic spinal metastases requiring surgical intervention (due to spinal instability, neurological deficit, or intractable pain); patients who underwent palliative surgery (e.g., decompression, fixation) for spinal metastases; and patients with complete preoperative clinical data, including Karnofsky Performance Status (KPS) scores. The exclusion criteria were: spinal metastases from a primary cancer other than lung cancer; patients who received nonoperative management for their spinal metastases; patients with incomplete medical records or missing baseline KPS data.

Surgical indications include spinal instability caused by pathological fractures or metastatic epidural spinal cord compression, decompressing the spinal canal, piecemeal resection of the tumor and internal fixation as necessary are the main palliative surgical procedures. All participants were followed up for 6 months (± 2 months), with follow-up information available for 128 patients. Follow-up was not possible for 18 patients due to the following reasons: 8 were unreachable, 6 had moved to other locations, and 4 were unwilling to participate in follow-up. The study was approved by the Ethics Committee of Peking University Third Hospital (approval number: M2023797) and was conducted in accordance with the Declaration of Helsinki. Informed consent was obtained from all study participants prior to the commencement of the research.

### 2.2. Data collection

Clinical information, including age, gender, clinical signs, radiological findings, histopathological results and occurrence of spine metastases, were systematically collected from the medical records. Preoperative pain and neurological status were measured using the Visual Analog Scale and the Frankel grade.^[9]^ The Spinal Instability Neoplasic Score,^[10]^ revised Tokuhashi score,^[7]^ and Epidural Spinal Cord Compression grade were assessed by 2 independent spine surgeons, and their average scores were recorded preoperatively. Quality of life was evaluated using the KPS before the operation, 1 week after the operation (± 3 days), and 6 months (± 2 months) after the operation. Participants were divided into 2 groups based on changes in KPS: the improvement group and the non-improvement group. In the improvement group, the KPS increased from the preoperative assessment to the follow-up time, while in the non-improvement group, the KPS remained the same or decreased. This dichotomization was chosen for its direct clinical relevance and interpretability. The goal was to identify factors predicting a tangible functional gain versus non-improvement (stabilization or decline) after a significant palliative intervention. In this high-risk population, defining a successful outcome as a distinct functional improvement provides a clear, albeit conservative, benchmark for evaluating the benefits of surgery.

### 2.3. Statistical analysis

The normality of data distribution was tested using the Shapiro–Wilk test. Categorical variables were analyzed using Chi-squared tests. Normally distributed variables were tested using Student *t* test, and non-normally distributed data were analyzed using the Mann–Whitney *U* test. All baseline clinical and demographic characteristics presented in Table 1, including preoperative Frankel grade, ambulatory status, and sphincter function, were first assessed in a univariate analysis. To further identify the clinical factors associated with improved quality of life, binary logistic regression analysis was conducted after adjusting for parameters with statistical significance in the univariate analysis. All descriptive statistics and parametric and nonparametric tests were performed using the software was SPSS software version 26 (IBM Corp., Armonk). For the 6-month follow-up analysis, mortality was treated as an outcome. Patients who died prior to the 6-month time point were included in the analysis and assigned to the “non-improvement group,” as death represents the definitive failure to achieve an improved quality of life.

**Table 1:** Clinical characteristics of the study participants and the results of the univariate analysis comparing groups with and without improved quality of life post-operation during short- and long-term assessments.

	Preoperation	Assessment 1 week post-operation		Assessment 6 months post-operation	
	Total (N = 128)	Non-improvement group (N = 26)	Improvement group (N = 102)	Non-improvement group (N = 16)	Improvement group (N = 92)
Male, n (%)*	72 (56.2)	8 (32.0)	63 (61.8)	9 (56.2)	53 (57.6)
Age (yr)	60 ± 10	60 ± 9	60 ± 11	63 ± 9	60 ± 10
BMI (kg/m^2^)	22.7 ± 3.3	22.9 ± 3.5	22.4 ± 2.2	23.0 ± 2.2	22.7 ± 3.4
Nonambulatory status preoperatively, n (%)	36 (28.1)	8 (30.7)	28 (27.5)	4 (25.0)	28 (30.4)
Preoperative bowel and bladder function, n (%)					
No impairment	105 (82.0)	19 (73.1)	86 (84.3)	14 (87.5)	76 (82.6)
Urinary impairment	11 (8.6)	2 (7.8)	9 (8.8)	1 (6.3)	7 (7.6)
Bowel impairment	5 (3.9)	1 (3.8)	4 (3.9)	1 (6.3)	5 (5.4)
Bowel and urinary impairment	7 (5.5)	2 (7.7)	5 (4.9)	0 (0.0)	4 (4.3)
Acute deterioration of neurological function, n (%)	39 (30.5)	11 (42.3)	27 (26.5)	5 (31.2)	28 (30.8)
Preoperative Frankel score, n (%)					
A	5 (3.9)	2 (7.7)	3 (2.9)	1 (6.3)	3 (3.3)
B	11 (8.6)	2 (7.7)	9 (8.8)	1 (6.3)	9 (9.8)
C	19 (14.8)	6 (23.1)	13 (12.7)	3 (18.8)	13 (14.1)
D	48 (37.5)	7 (26.9)	41 (40.2)	5 (31.3)	31 (33.7)
E	45 (35.2)	9 (34.6)	36 (35.3)	6 (37.5)	36 (39.1)
Total number of bone metastases, median (interquartile)*^,^**	3 (2, 5)	6 (5, 8)	3 (2, 4)	4 (2, 5)	3 (2, 5)
Number of spine metastases, median (interquartile)*^,^**	2 (1, 3)	4 (3, 5)	2 (1, 2)	2 (1, 3)	2 (1, 3)
Visceral metastasis, n (%)	25 (19.5)	7 (26.9)	18 (18.2)	2 (12.5)	19 (20.7)
Pulmonary metastasis, n (%)	71 (55.5)	13 (50.0)	58 (58.0)	9 (56.2)	56 (60.9)
Pathological fracture, n (%)	68 (53.1)	11 (42.3)	57 (55.9)	7 (43.8)	53 (57.6)
Preoperative VAS score, median (interquartile)	7 (5, 8)	6 (4, 8)	7 (5, 8)	7 (6, 8)	7 (5, 8)
Preoperative KPS, median (interquartile)	60 (40, 70)	60 (40, 80)	60 (40, 70)	60 (40, 70)	60 (40, 70)
Preoperative SINS score, n (%)					
0–6	4 (3.1)	2 (7.7)	2 (2.0)	1 (6.3)	3 (3.3)
7–12	86 (67.2)	17 (65.4)	69 (67.6)	13 (81.3)	60 (65.2)
13–18	38 (29.7)	7 (26.9)	31 (30.4)	2 (12.5)	29 (31.5)
Preoperative ESCC Grade, n (%)					
Grade 0	2 (1.6)	1 (3.8)	1 (1.0)	1 (6.3)	1 (1.1)
Grade 1	40 (31.2)	5 (19.2)	35 (34.3)	8 (50.0)	27 (29.3)
Grade 2	53 (41.4)	13 (50.0)	40 (39.2)	4 (25.0)	37 (40.2)
Grade 3	33 (25.8)	7 (26.9)	26 (25.5)	3 (18.8)	27 (29.3)
Revised Tokuhashi score (median, interquartile)	6 (4, 7)	5 (4, 7)	6 (4, 7)	6 (4, 7)	6 (4, 7)
surgical approach					
Anterior	27 (21.1)	5 (19.2)	22 (21.6)	4 (25.0)	17 (18.5)
Posterior	101 (78.9)	21 (80.8)	80 (78.4)	12 (75.0)	75 (81.5)
Pathology of metastatic lesions					
Squamous cell carcinoma	21 (16.7)	4 (15.4)	17 (17.0)	2 (12.5)	14 (15.2)
Adenocarcinoma	87 (69.0)	16 (61.5)	71 (71.0)	10 (62.5)	68 (73.9)
Small cell lung cancer	9 (7.1)	2 (7.7)	7 (7.0)	2 (12.5)	6 (6.5)
Others	9 (7.1)	4 (15.4)	5 (5.0)	0 (0.0)	4 (4.3)
Nonambulatory status 6 months postoperatively, n (%)*^,^**	13 (10.2)	9 (34.6)	4 (4.5)	1 (6.3)	12 (10.7)
Postoperative VAS score, median (interquartile)					
One-week post-operation	4 (2, 4)	4 (2, 5)	4 (2, 4)	2 (2, 3)	4 (2, 4)
Six months post-operation	0 (0, 2)	0 (0, 3)	0 (0, 2)	/	0 (0, 2)
Postoperative KPS, median (interquartile)					
One-week post-operation*	70 (50, 80)	50 (30, 80)	75 (60, 80)	75 (60, 80)	70 (50, 80)
Six months post-operation	90 (80,100)	90 (40, 100)	90 (80, 100)	/	100 (80, 100)
Combination of other treatment, n (%)					
Radiotherapy	61 (47.7)	13 (50.0)	48 (47.1)	6 (37.5)	44 (47.8)
Chemotherapeutic drugs and targeted therapies	84 (65.6)	18 (69.2)	66 (64.7)	9 (56.3)	59 (64.1)

*Note:* Normally distributed data are expressed as mean ± SD, while non-normally distributed data are presented as median (25th percentile, 75th percentile). Qualitative information was depicted as the number of cases (percentage). Participants in the non-improvement group at the 6-month assessment were missing VAS and KPS scores.

BMI = body mass index, CI = confidence interval, ESCC = Epidural Spinal Cord Compression, KPS = Karnofsky Performance Status, SINS = Spine Instability Neoplastic Score, VAS = Visual Analog Scale.

* Comparison between the groups with and without improved quality of life post-operation at 1 week showed a statistically significant difference (*P* < .05).

** Comparison between the groups with and without improved quality of life post-operation at 6 months follow-up showed a statistically significant difference (*P* < .05).

## 3. Results

### 3.1. Clinical characteristics of the participants

A total of 128 participants were recruited and followed in the study, consisting of 72 males and 56 females, with a mean age of 60 ± 10 years. Before the operation, 28.1% of the participants were nonambulatory. Additionally, 8.6% had urinary impairment, 3.9% had bowel impairment, and 5.5% had both bowel and urinary impairments. Furthermore, 30.5% of the participants experienced acute deterioration of neurological function (<48 hours). The distribution of Frankel scores among the participants was as follows: 3.9% scored A, 8.6% scored B, 14.8% scored C, 37.5% scored D, and 35.2% scored E.

The median number of spinal metastases sites was 2 (interquartile range 1–3), while the median number of total bone metastases, including both spinal and non-spinal sites, was 3 (interquartile range 2–5). Additionally, 19.5% had metastases in other visceral organs, and 55.5% had pulmonary metastases. The median preoperative KPS score was 60 (interquartile range 40–70), and the median Tokuhashi score was 6 (interquartile range 4–7). The median preoperative Visual Analog Scale score was 7 (interquartile range 5–8). According to the Spinal Instability Neoplasic Score, 3.1% of patients had stable lesions (scores 0–6), 67.2% had potentially unstable lesions (scores 7–12), and 29.7% had unstable lesions (scores 13–18). For the Epidural Spinal Cord Compression, 1.6% were Grade 0, 31.2% were Grade 1, 41.4% were Grade 2, and 25.8% were Grade 3. Adenocarcinoma was the most common type of pathology observed (69.0%). Additionally, 78.9% of the patients underwent a posterior surgical approach (Table 1).

### 3.2. Factors related to short-term quality of life improvement post-operation

In assessing the quality of life 1-week post-surgery, a higher percentage of males reported improvement than females (61.8% vs 32.0%, *P* < .05). Participants who experienced an improvement in quality of life had fewer bone metastatic sites both in the whole body and in the spine: 3 (interquartile range: 2–4) vs 6 (interquartile range: 5–8) for total bone metastases, and 2 (interquartile range: 1–2) vs 4 (interquartile range: 3–5) for spinal metastases (*P* < .05 for both comparisons). In multivariate analysis, male sex was associated with improved quality of life (odds ratio [OR] = 0.42, 95% confidence interval [CI] [0.716–0.962]), and the number of total bone metastatic sites was negatively associated with improved quality of life (OR = 3.66, 95% CI [1.55–8.67]).

### 3.3. Factors related to long-term quality of life improvement post-operation

In the 6-month quality of life assessment, 16 participants were lost to follow-up. A comparison between the entire cohort and those lost to follow-up indicated no significant differences (Table 2 and Table S1, Supplemental Digital Content, https://links.lww.com/MD/Q560). The long-term quality of life assessment showed no notable differences based on sex. However, participants who experienced improved quality of life had a lower number of total bone metastases and spinal metastases. Specifically, those with improved quality of life had a median of 3 total metastases (interquartile range: 2–5) compared to 4 (interquartile range: 2–5) in those without improvement, and a median of 2 spinal metastases (interquartile range: 1–3) versus 2 (interquartile range: 1–2) (*P* < .05 for both comparisons). The logistic regression analysis revealed that a higher number of total bone metastatic sites was negatively associated with improved quality of life, with an OR of 1.94 and a 95% CI of [1.05–3.59].

**Table 2:** Factors associated with improved quality of life in both short- and long- term follow-up according to multivariable logistic regression.

	Assessment 1 week post-operation		Assessment 6 months post-operation	
	OR	95% CI	OR	95% CI
Marital status				
Female	Referent		/	/
Male	0.42	0.12, 0.66	/	/
Number of bone metastases	3.66	1.55, 8.67	1.94	1.05–3.59
Number of spine metastases	1.72	0.79, 3.72	0.82	0.52–1.29

Multivariable logistic regression including all variables before surgery with *P* < .10 in univariate analysis.

CI = confidence interval, OR = odds ratio.

## 4. Discussion

It is crucial to interpret the findings of this study within the broader clinical context of palliative spinal surgery. The decision to operate is often driven by urgent and compelling indications, such as treating or preventing catastrophic neurological deficits from spinal cord compression, stabilizing pathological fractures to allow mobilization, and alleviating intractable oncologic pain. These interventions are fundamental to patient care and can be prerequisites for enabling patients to receive potentially life-prolonging systemic therapies. Our study does not challenge the necessity of surgery for these critical indications. Rather, our findings are intended to supplement the decision-making process by providing prognostic information specifically related to postoperative functional improvement, a key component of quality of life. For a patient with a high metastatic burden, surgery may still be the best option to preserve neurological function, but our data suggest that clinicians and patients should have a realistic conversation about the likelihood of achieving a significant gain in overall independence. This helps align the goals of a high-risk intervention with a patient’s individual circumstances and overall disease trajectory.

Lung cancer is one of the most common malignant tumors, with many patients developing spinal metastases as the disease progresses, resulting in skeletal-related events. Patients with spine metastases from lung cancer encounter various challenges related to physical, functional, and psychosocial factors. While surgery is a practical approach for decompression, stabilization and reducing tumor burden, it can also be a double-edged sword for these end-stage cancer patients. Although some previous studies have reported on life expectancy following surgery, few have focused on quality of life. In this retrospective study of prospectively collected data, we present findings from a relatively large sample, revealing that the number of tumor metastases in both the spine and other bone sites is negatively associated with postoperative quality of life.

Surgery combined with adjuvant radiotherapy could lead to more significant improvements in neurological function than radiotherapy alone, which has substantial implications for spinal oncology surgery.^[11]^ However, these surgical procedures often carry a high risk of perioperative mortality and morbidity.^[12,13]^ In cases of symptomatic spinal metastases due to lung cancer, where the treatment intent is often palliative, quality of life becomes paramount. Patient-reported outcome measures are crucial endpoints that should be prioritized in clinical research and practice.

Similar to previous studies,^[2,4]^ our analysis of baseline participant characteristics revealed that individuals with lung cancer metastases requiring spinal surgery are in deplorable condition. Specifically, 28.1% had lost their ability to ambulate, 18.0% experienced urinary and/or bowel impairment, 30.5% had acute deterioration of neurological function, 19.5% had visceral metastasis, and 55.5% had pulmonary metastasis. Among all solid cancers, lung cancer has the worst prognosis for patients with spinal metastases. These clinical signs indicate a highly vulnerable and complex group of end-stage cancer patients. The risks associated with surgery in this population are particularly concerning for patients with limited life expectancy, as its side effects may outweigh the potential benefits of surgery. Although progress has been made in the management of spinal metastases from lung cancer,^[14]^ the shortened life expectancy underscores the importance of considering the patient as a whole when making clinical decisions, particularly regarding quality of life.

Sex differences in treatment outcomes are influenced not only by medical issues but also by psychological and social factors, which are essential contributors to the quality of life for patients after surgery. A systematic review that analyzed surgical outcomes in male and female patients undergoing orthopedic surgery found that approximately 77% of the studied research reported sex/gender-related differences in postoperative outcomes, with over 55% indicating worse postoperative results for female patients in terms of pain, disability, health-related quality of life, and complications.^[15]^ Additionally, another study noted that females may experience increased and prolonged opioid use compared to males after spinal surgery.^[16]^ While sex is a non-modifiable demographic factor, its value in this context is as a risk stratification tool. Identifying patient subgroups at higher risk for suboptimal outcomes is crucial for personalizing care. This finding does not necessarily imply a direct biological cause, but rather may reflect a complex interplay of physiological, psychological, and social factors that differ between sexes. For instance, studies have reported sex-based differences in pain perception, responses to analgesia, and higher rates of postoperative anxiety or depression in female patients, which could all negatively impact short-term recovery and perceived quality of life. Therefore, this result should empower clinicians to provide targeted supportive care. For female patients undergoing this major surgery, this could include more aggressive multimodal pain management, preemptive mental health support, and earlier mobilization with physical therapy. Our finding highlights that female patients may require more intensive, multifaceted attention in the immediate postoperative period to optimize their recovery trajectory.

The involvement of multiple vertebrae and other bone sites typically indicates a more advanced stage of disease and may significantly reduce patient survival times. According to a recently published systematic review of 14 studies, the number of bone metastasis sites was associated with prognosis.^[6]^ Additionally, a meta-analysis comprising 43 studies on spinal metastases from various cancer origins found that the presence of multiple bone metastases (more than 2 sites) and multiple spinal metastases (more than 3 sites) were associated with an unfavorable prognosis.^[17]^ However, the novelty of our finding is not in reconfirming this link to prognosis, but in identifying the number of total bone metastases as a specific, independent predictor for the postoperative functional outcome. While survival is a critical consideration, the primary goal of palliative surgery is often to improve the patient’s remaining quality of life. Our study provides evidence that a high metastatic burden makes a tangible improvement in functional status (as measured by KPS) less likely. This distinction is clinically vital. It allows clinicians to use a simple preoperative factor to help set realistic expectations. For a patient with many bone metastases, the surgical goal may be more appropriately framed as preserving current neurological function and preventing further decline, rather than achieving a significant improvement in overall functional independence. This reframing is a cornerstone of effective shared decision-making in palliative oncology.

Our study does have limitations. Firstly, it is a single-center study. However, the relatively large sample size may compensate for this limitation. Given that spinal metastases due to lung cancer present a challenging clinical situation and only an experienced specialized spine surgery team can perform such procedures, we believe our participants were somewhat representative of the broader population.

Secondly, the follow-up period was only 6 months. This time point was chosen because it represents a clinically meaningful milestone for assessing the durability of a palliative intervention. While the literature often cites a median survival of approximately 6 months for the general population of lung cancer patients with spinal metastases, it is crucial to note that our surgical cohort represents a selected group with likely better baseline functional status and more manageable disease, leading to a longer actual survival than the general average. This selection bias explains why a significant portion of our cohort was available for follow-up. Furthermore, with the continuous improvement in systemic therapies, the median survival itself is increasing. Understanding the durability of functional improvement in these longer-term survivors is a critical unanswered question. Therefore, we strongly recommend that future prospective studies be designed with extended follow-up points (e.g., at 12 and 24 months). This is not only realistic but essential for capturing the complete long-term trajectory of quality of life, which is becoming increasingly relevant in the modern era of oncology.

Thirdly, a significant limitation of our study is its reliance solely on the KPS as a measure of quality of life. The choice to use KPS was pragmatic, driven by its consistent and routine collection in our clinical practice throughout the long study period, making it the most complete dataset available for this retrospective analysis. However, we acknowledge that KPS is fundamentally a measure of functional performance and does not encompass the multidimensional nature of quality of life. It does not capture crucial patient-reported domains such as detailed pain levels, emotional well-being, or social functioning, which are vital to a holistic understanding of a patient’s state. Furthermore, as a clinician-assessed score, it may not fully reflect the patient’s subjective experience, which is better captured by patient-reported outcome measures like the EQ-5D or the European Organisation for Research and Treatment of Cancer QLQ-C30. Future prospective studies should undoubtedly incorporate these more comprehensive assessment tools to provide a more complete evaluation of the quality of life for these patients.

Fourthly, the long study period (2009–2020) and the retrospective nature of the study present 2 major limitations regarding confounding variables. On the one hand, this period covers an era of significant evolution in systemic treatments for lung cancer, including the introduction of targeted therapies and immunotherapy. We were unable to collect detailed, consistent data on the specific systemic regimens patients received, and this represents a major unmeasured confounder. These advanced therapies undoubtedly influenced both overall survival and quality of life, and their omission from our model is a significant limitation. On the other hand, while we controlled for key demographic and metastatic factors, other unmeasured confounders such as baseline nutritional status or psychosocial factors could also influence outcomes. Future prospective studies are essential to control for the crucial variable of systemic therapy.

Fifthly, the long 11-year enrollment period spans a period of revolutionary change in the management of metastatic lung cancer. The introduction and widespread adoption of targeted therapies for driver mutations (e.g., EGFR, ALK) and immune checkpoint inhibitors have dramatically improved survival outcomes for select patients. Furthermore, advances in surgical techniques, anesthetic management, and the integration of Stereotactic Body Radiation Therapy have altered the perioperative landscape. This evolution creates considerable heterogeneity within our cohort. A patient treated in 2010 had a different prognosis and treatment pathway than an identical patient treated in 2020. We were unable to stratify our analysis by treatment era or specific systemic therapy due to the retrospective nature and sample size constraints. This unmeasured confounding undoubtedly influences our results and limits the direct applicability of our Cohort absolute outcomes to contemporary practice. Despite this major limitation, we contend that the study’s central finding (the negative predictive power of a high metastatic burden on functional recovery) remains relevant. The total number of bone metastases serves as a strong proxy for the overall aggressiveness of the disease and the patient’s physiological reserve. This fundamental relationship is likely to persist even with improved systemic treatments. Therefore, while the overall prognosis for patients has improved, the relative impact of a high disease burden on the ability to recover from a major surgical intervention likely remains a critical and durable consideration for clinicians today.

Finally, and perhaps most importantly, our analysis does not account for the status of systemic disease progression at the 6-month follow-up, which is a powerful confounder for quality of life. A patient’s functional status at 6 months is profoundly influenced by their overall cancer trajectory. A decline in KPS could be entirely due to the progression of visceral metastases, uncontrolled pain from other sites, or general cancer-related cachexia and fatigue, even if the spinal surgery was technically successful in achieving local decompression and stability. Because this study’s goal was to identify preoperative predictors, our key finding (that a high baseline metastatic burden predicts a poor functional outcome) may be partially explained by this phenomenon. A higher number of bone metastases at the time of surgery likely serves as a strong proxy for more aggressive disease biology and a higher probability of rapid systemic progression. Future prospective studies should be designed to co-register systemic disease status at all follow-up points to successfully disentangle the specific effects of the surgical intervention from the powerful confounding effects of the underlying cancer’s progression.

## 5. Conclusion

This study’s findings provide practical guidance for a more personalized and patient-centered approach to the surgical management of spinal metastases from lung cancer. We recommend the following:

1. Use metastatic burden to guide patient selection and manage expectations: The total number of bone metastatic sites should be used as a key criterion in preoperative counseling. For patients with a high metastatic burden, clinicians should be cautious when promising significant functional improvement. The primary goal of surgery in this subgroup may be more appropriately defined as the preservation of neurological function and spinal stability, rather than a substantial improvement in overall quality of life. This realistic framing is essential for shared decision-making.
2. Implement targeted perioperative care for at-risk subgroups: Our finding that female patients are at higher risk for poor short-term outcomes calls for a proactive, personalized care strategy. We recommend implementing enhanced supportive care protocols for female patients, which could include aggressive multimodal pain control, early and intensive rehabilitation, and proactive psychosocial support to mitigate this risk.

In conclusion, by moving beyond general survival prognostication, our study provides clinicians with specific, preoperative factors (metastatic burden and sex) to better predict functional outcomes. This allows for more nuanced patient counseling and the development of tailored care pathways designed to optimize the quality of life for these vulnerable individuals.

## Acknowledgments

The authors would like to thank all the participants and their relatives for their willingness and support in participating in this study.

## Author contributions

**Conceptualization:** Yan Li, Fangzhi Liu.

**Data curation:** Yan Li, Fangzhi Liu.

**Formal analysis:** Yan Li, Fangzhi Liu.

**Funding acquisition:** Yan Li, Fangzhi Liu.

**Investigation:** Yan Li, Fangzhi Liu.

**Methodology:** Yan Li, Fangzhi Liu.

**Project administration:** Yan Li, Fangzhi Liu.

**Resources:** Zhongjun Liu, Xiaoguang Liu, Hua Zhou, Xiao Liu, Yanchao Tang, Panpan Hu.

**Software:** Yan Li, Fangzhi Liu.

**Supervision:** Feng Wei.

**Validation:** Yan Li, Fangzhi Liu.

**Visualization:** Yan Li, Fangzhi Liu.

**Writing – original draft:** Yan Li, Fangzhi Liu.

**Writing – review & editing:** Yan Li, Fangzhi Liu.

## Supplementary Material


