# Supplementary material for: Factors associated with postoperative quality of life in patients with spinal metastases from lung cancer
Source: Medicine (Baltimore). 2025 Nov 7;104(45):e45359. doi: 10.1097/MD.0000000000045359 (PMC12599724; doi:10.1097/MD.0000000000045359)
Supplement: Supplementary file 1 [file medi-104-e45359-s001.docx]

| Supplementary Table 1. Comparison of baseline characteristics between all participants and those lost to follow-up at six months | | |
| --- | --- | --- |
|  | Total Participants  (N=128) | Participants Lost to Follow-Up at Six Months  (N=16) |
| Male, n (%) | 72 (56.2) | 9 (56.2) |
| Age, years | 60±10 | 57±9 |
| BMI, kg/m^2^ | 22.7±3.3 | 22.6±3.6 |
| Non-Ambulatory Status Preoperatively, n (%) | 36 (28.1) | 4 (25.0) |
| Preoperative Bowel and Bladder Function, n (%) |  |  |
| No Impairment | 105 (82.0) | 14 (87.5) |
| Urinary Impairment | 11 (8.6) | 1 (6.3) |
| Bowel Impairment | 5 (3.9) | 1 (6.3) |
| Bowel and Urinary Impairment | 7 (5.5) | 0 (0.0) |
| Acute Deterioration of Neurological Function, n (%) | 39 (30.5) | 5 (31.2) |
| Preoperative Frankel score, n (%) |  |  |
| A | 5 (3.9) | 1 (6.2) |
| B | 11 (8.6) | 1 (6.2) |
| C | 19 (14.8) | 3 (18.8) |
| D | 48 (37.5) | 5 (31.2) |
| E | 45 (35.2) | 6 (37.5) |
| Total Number of Bone Metastases, median (interquartile) | 3 (2, 5) | 3(2, 5) |
| Number of Spine Metastases, median (interquartile) | 2 (1, 3) | 3 (1,3) |
| Visceral Metastasis, n (%) | 25 (19.5) | 2 (12.5) |
| Pulmonary Metastasis, n (%) | 71 (55.5) | 9 (56.2) |
| Pathological Fracture, n (%) | 68 (53.1) | 7 (43.8) |
| Preoperative VAS score, median (interquartile) | 7(5, 8) | 7 (6, 8) |
| Preoperative KPS, median (interquartile) | 60 (40, 70) | 60 (40, 70) |
| Preoperative SINS score, n (%) |  |  |
| 0-6 | 4 (3.1) | 1 (6.3) |
| 7-12 | 21 (16.4) | 2 (12.5) |
| 13-18 | 103 (80.5) | 13 (81.2) |
| Preoperative ESCC Grade, n (%) |  |  |
| Grade 1 | 2 (1.6) | 1 (6.3) |
| Grade 2 | 40 (31.2) | 8 (50.0) |
| Grade 3 | 53 (41.4) | 4 (25.0) |
| Grade 4 | 33 (25.8) | 3 (18.7) |
| Pathology of Metastatic Lesions |  |  |
| Squamous Cell Carcinoma | 21 (16.7) | 2 (12.5) |
| Adenocarcinoma | 87 (69.0) | 10 (62.5) |
| Small Cell Lung Cancer | 9 (7.1) | 2 (12.5) |
| Others | 9 (7.1) | 2 (12.5) |
| Revised Tokuhashi score, (median, interquartile) | 6 (4, 7) | 6 (4, 7) |
| Combination of Other Treatment, n (%) |  |  |
| Radiotherapy | 61 (47.7) | 7 (43.8) |
| Antitumor Drug Therapy | 84 (65.6) | 9 (56.3) |
| *Note:* Normally distributed data are expressed as mean ± SD, while non-normally distributed data are presented as median (25th percentile, 75th percentile). Qualitative information was depicted as the number of cases (percentage).  *Abbreviation*: BMI, body mass index; VAS, Visual Analog Scale; KPS, Karnofsky Performance Status; SINS, Spine Instability Neoplastic Score; ESCC, Epidural Spinal Cord Compression. | | |
